# Smartphone Apps for Managing Antithrombotic Therapy: Scoping Literature Review

**DOI:** 10.2196/29481

**Published:** 2022-06-21

**Authors:** Friederike Praus, Bartosz Krzowski, Tabea Walther, Christian Gratzke, Paweł Balsam, Arkadiusz Miernik, Philippe Fabian Pohlmann

**Affiliations:** 1 Department of Urology Faculty of Medicine University of Freiburg-Medical Centre Freiburg Germany; 2 First Department of Cardiology Medical University of Warsaw Warsaw Poland

**Keywords:** anticoagulation, mobile app, telehealth, telemedicine, mHealth, smartphone, educational apps, digital tools, physician support

## Abstract

**Background:**

Antithrombotic therapy is complex and requires informed decisions and high therapy adherence. Several mobile phone apps exist to either support physicians in the management of antithrombotic therapies or to educate and support patients. For the majority of these apps, both their medical evidence and their development background are unknown.

**Objective:**

This review aims to investigate the available literature describing high-quality apps for managing antithrombotic therapy based on professional scientific information.

**Methods:**

Keywords and Medical Subject Heading terms were used to search MEDLINE via PubMed and Ovid between December 2019 and January 2022. Inclusion criteria were the availability of full text and publications in the English language. Apps that solely focused on atrial fibrillation were excluded. Qualitative findings were thematically synthesized and reported narratively.

**Results:**

Out of 149 identified records, 32 were classified as eligible. We identified four groups: (1) apps for patients supporting self-management of vitamin K antagonists, (2) apps for patients increasing therapy adherence, (3) educational apps for patients, and (4) apps for physicians in supporting guideline adherence.

**Conclusions:**

Throughout the evaluated data, patients from all age groups receiving antithrombotic drugs expressed the desire for a digital tool that could support their therapy management. In addition, physicians using mobile guideline-based apps may have contributed to decreased adverse event rates among their patients. In general, digital apps encompassing both user-friendly designs and scientific backgrounds may enhance the safety of antithrombotic therapies. However, our evaluation did not identify any apps that addressed all antithrombotic drugs in combination with perioperative stratification strategies. Currently, strict regulations for smartphone apps seem to negatively affect the development of new apps. Therefore, new legal policies for medical digital apps are urgently needed.

## Introduction

Antithrombotic therapy, including both anticoagulation and platelet aggregation inhibition, is a common therapy for the treatment and prevention of atrial fibrillation (AFib)–related thromboembolic events [1], venous thromboembolism [2], and coronary artery disease [3]. European guidelines recommend oral anticoagulation (OAC) for most patients with AFib and platelet aggregation inhibitors for every patient diagnosed with coronary artery disease without contraindications [3,4]. Although AFib guidelines do not recommend OAC for low-risk patients, 75% of these patients receive antithrombotic therapy. On the other hand, 12% of patients at high risk for thromboembolic events do not receive adequate antithrombotic therapy [5]. The increasing number of new agents and drug combinations, as well as expanding indications for antithrombotic therapy, might contribute to inadequate management, putting these patients at risk for adverse events. Inadequate treatment might result in both bleeding and insufficient protection from thromboembolic events [6]. Several approaches were studied over the past years. However, mobile apps are constantly gaining in interest and also in clinical utility [7].

In 2021, the number of smartphone subscriptions worldwide reached the threshold of 6 billion, and that number is expected to reach 7.5 billion in 2026 [8]. Smartphones incorporate various hardware and software features. Furthermore, they enable wireless connectivity. In addition, the user can adapt the phone’s software functions by individually installing apps. Apps are computer programs that run on the smartphone’s mobile operating system (eg, Android and iOS).

The objective of this paper was to provide a comprehensive review of antithrombotic therapy apps that have been scientifically evaluated. Summarizing these results, our paper also offers suggestions for the future implementation of comparable apps.

## Methods

We searched MEDLINE via PubMed and Ovid for predefined Medical Subject Headings and keywords in titles and abstracts. Searches in PubMed and Ovid were performed between December 8, 2019, and January 25, 2022, by an expert in the field of literature searching. The detailed search strategies are available in Multimedia Appendix 1. Additionally, we searched for publications about relevant apps.

Titles and abstracts were screened independently for eligibility by three authors (FP, BK, and TW). In the case of a disagreement, a fourth author (AM) was consulted. Full-text screening was performed independently by three authors (FP, BK, and TW).

Publications about smartphone apps concerning any aspect of antithrombotic therapy (eg, management, guidelines, risk assessment, or education) were considered eligible. Apps in this context were defined as smartphone software apps that must be installed on the device. Other features and hardware components were not defined as apps and, therefore, were among the exclusion criteria. Reviews, clinical studies, and protocols were included if their full text was available in English. “Epub ahead of print” articles were also included. There was no limit regarding publication date.

Data were collected from the publications by two authors (FP and TW). Information about the type of publication (eg, development of the app, user evaluation, and feasibility study); the name, target group, and aim of the app; study results; study funding; and country where the study was performed or where the app was developed were collected. Based on the main target group and the main aim of the app, the apps were assigned to groups. If not stated in the record, commercial availability of the apps was assessed by browsing Google Play and the Apple App Store.

Due to the heterogeneity of the publications, no meta-analysis or additional analyses were performed. Extracted data were analyzed descriptively.

## Results

### Overview

**Figure 1:**
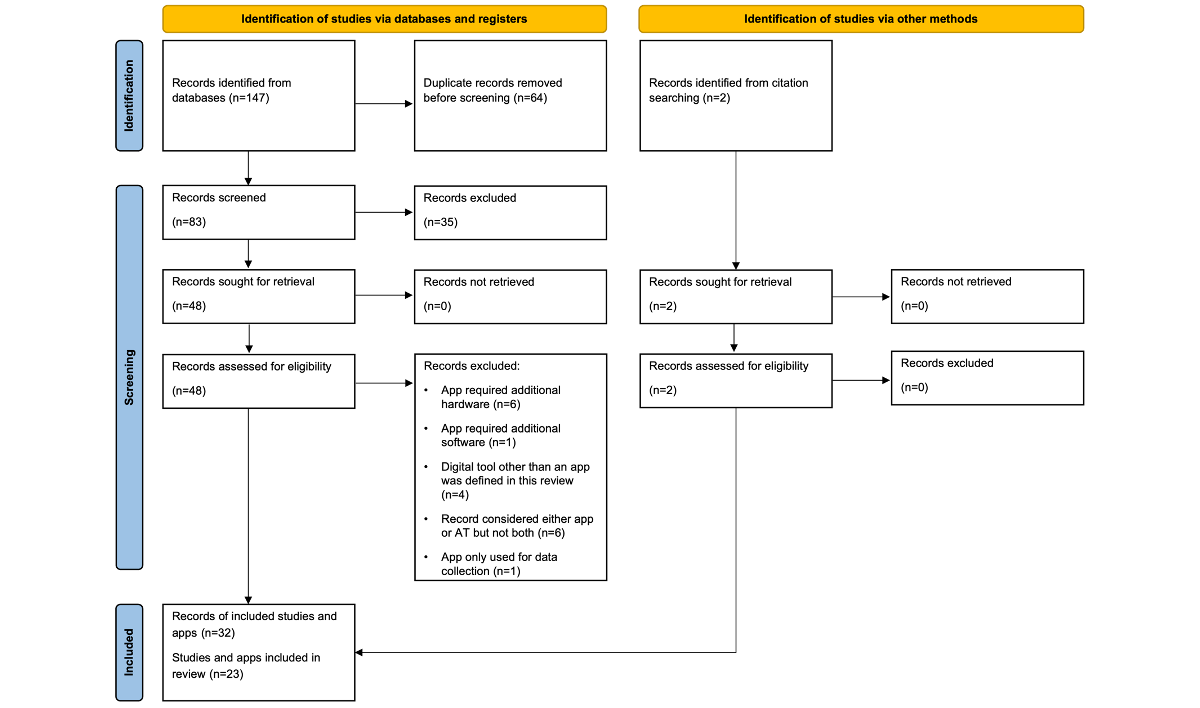
PRISMA (Preferred Reporting Items for Systematic Reviews and Meta-Analyses) flow diagram showing the number of records identified, records included, and records excluded with the reasons for exclusion. AT: antithrombotic therapy.

Our search revealed 149 records (147 from the databases and 2 from citation searching); this number was reduced to 32 records by excluding duplicates and applying inclusion and exclusion criteria, as shown in Figure 1. These 32 records were included in the qualitative synthesis. The 32 records reported on 23 different apps or app projects. Among them, 10 (31%) reported on the development of an app and 15 (47%) studied the efficacy of an app. Out of 32 records, 8 (25%) reported on evaluation of an app, another 4 (13%) were study protocols or were associated with a protocol, and 2 (6%) reported on app distribution and user statistics. Some reports covered more than one of these aspects. Only 1 (3%) report was a review. We identified four groups of smartphone apps concerning antithrombotic therapy: (1) apps for patients supporting self-management of vitamin K antagonists (VKAs), (2) apps for increasing patient treatment adherence, (3) apps for patient education, and (4) apps for supporting physicians with decision-making and guideline adherence. A comprehensive synthesis of the results is shown in Multimedia Appendix 2.

### Smartphone Apps for Patients Managing VKA Therapy

A report on the development and user evaluation of the app Warfarin Guide discussed a user-centered approach to design the app for patients taking warfarin, a VKA, to monitor their international normalized ratio (INR) measurements. Additionally, the app gives recommendations on warfarin dosages. After a co-design process, several usability tests were performed before app iteration [9,10]. In a study with 13 patients taking warfarin, a mean usability score of 85.0 (out of 100) was achieved based on the System Usability Scale [10]. A randomized controlled trial (RCT) validating the efficacy and effectiveness of Warfarin Guide was proposed. There was no funding declared.

An interdisciplinary team of computer scientists and cardiologists tracked INR measurements and recommended a VKA dosage from a technical point of view in an app (not named) based on two different machine learning algorithms [11]. A physician may access their patients’ values via a web interface. Automated warnings are sent to the physician in case of threatening INR values. There was no funding reported.

The app Anticlot Assistant also gives dosage recommendations to patients taking warfarin based on INR measurements and a predefined target INR. Compliance with the app and time spent within the therapeutic range were evaluated prospectively in 30 patients. A positive prognostic factor for good compliance was having received an education of greater than 6 years (odds ratio 8.4; *P*=.03). Patients with good compliance spent significantly more time within the therapeutic range (mean 65.6%, SD 25.0% vs mean 40.0%, SD 21.0%; *P*=.009). The project received a grant from the China National Natural Science Foundation [12].

The Chinese app named XY was developed to aid patients in dosing warfarin [13]. Against the backdrop of the COVID-19 pandemic and associated limited access to professional INR monitoring, the app aims to improve the amount of time spent within the therapeutic range. Patients submit their self-measured INR to the app and receive a dosing recommendation. Contact is made by medical professionals in predefined conditions (eg, INR considerably out of target range or bleeding). The authors of the report presented the protocol of the validation study comparing users of the app against patients in the control group in an RCT. Enrollment began in March 2021. The study is sponsored by the Chinese Medical Board.

The Alfalfa app was developed by a multidisciplinary team and focuses on point-to-point remote monitoring of warfarin therapy [14]. The app is divided into a patient terminal and a medical staff terminal. Patients must provide necessary information about their medical history. After submitting their current INR and warfarin dose to their assigned doctor, they receive a response on dose adjustment and the suggested date of their next blood test. The app also includes reminder functions; access to a community, where patients can share their experiences; and an educational subsection. The usability and learnability of Alfalfa was evaluated in a retrospective study with 26 users by the System Usability Scale. The patient terminal and medical terminal reached scores of 61.8 and 82.7 (out of 100), respectively. The need for improving the usability of the patient terminal and the ease of learning for both terminals became evident. The Alfalfa app runs on the platform WeChat, a product of the Chinese telecommunication enterprise Tencent. According to the authors, reasons for choosing this platform included high development costs and long reviewing periods for apps at the Apple App Store and for Android apps at Google Play.

Additionally, Alfalfa was tested in a retrospective, observational cohort study with 824 patients over 3 years to evaluate the effectiveness and safety of warfarin management via the Alfalfa app [15]. Clinical outcomes of patients using Alfalfa to adjust their warfarin dose and those of patients who attended regular hospital visits were compared. Alfalfa proved to be more effective than regular hospital visits in helping patients spend more time within the therapeutic range (79.35% vs 52.38%; *P*<.001), experience fewer major bleeding events (0.5% vs 3.0%; *P*=.005), and experience fewer warfarin-related emergency hospital admissions (0.2% vs 3.0%; *P*=.001).

The Yixing app was developed by hospital pharmacists who specialized in anticoagulation therapy. It includes functions such as reminders for drug intake, a personal health record, an educational program to raise the patient’s awareness, and online counseling. The authors conducted a prospective study with 100 patients who had received a valve replacement; patients were distributed equally into an intervention group, who were using the Yixing app after receiving app-based training, and a control group, who would receive oral medication training but no further support after being discharged from the hospital [16]. The results revealed that using the app could increase the patient’s awareness and days spent within the therapeutic range but did not have an influence on the number of days that warfarin was taken correctly or the incidence of anticoagulation-related complications.

### Smartphone Apps for Educating Patients

In the systematic review conducted by Jang [17], the focus was on educational programs. A total of 12 studies were included in the analysis. The author focused on patients taking VKA in a hospital setting. Some of the solutions that were described were telephone-based programs. Three of the papers were described in our analysis as well [18-20]. The author concluded that mobile health (mHealth) apps improve adherence and patient’s knowledge, but that bigger studies are required.

A group of cardiologists reported on an app (not named) that was developed by a multidisciplinary team based on current scientific literature. Inpatients diagnosed with AFib were given this app to educate themselves on thromboembolic and bleeding risk in AFib and on different treatments to improve shared decision-making [20]. The patient’s knowledge on AFib was tested with a 20-item questionnaire before and after using the app. It showed a significant increase from a mean number of 4.7 (SD 1.8) to 7.2 (SD 1.0) correct answers (*P*<.001). However, perception of individual risks did not change significantly. The study was funded by internal budgets.

A similar approach was taken by a Philippine team. Based on focus group discussions and a literature review, they developed an app (not named) to support shared decision-making for or against OAC [21]. AFib patients use the app as part of their medical consultation to be educated about AFib and the different options regarding OAC. A pilot test with 37 patients showed a significant increase in AFib knowledge of 5 points (24-point knowledge tool; *P*<.001) and a significant decrease in decisional conflict of 35 points (100 point–scaled Ottawa PDA [patient decision aid] Decisional Conflict Scale; *P*<.001). Acceptance of the app by 37 patients and 30 physicians was mostly good to very good (92%-100% of patients in different categories; 67%-97% of physicians in different categories). The development of the app was funded by the pharmaceutical company Pfizer.

An interdisciplinary team reported on the development and evaluation of the Mobile Applications for Seniors to Enhance Safe Anticoagulation Therapy (MASS) [19,22]. MASS provides education on different anticoagulation therapies, their risks, and food recommendations for patients on VKA. Diary functions for INR, blood values, symptoms of bleeding, and reminders for medications are also included. In a co-design process, participants described their medication self-management and experience with digital health tools [22]. A feasibility study with 18 patients showed significant improvement of anticoagulation knowledge (*P*=.007). Other outcomes, such as therapy satisfaction, therapy adherence, and depressive or anxiety symptoms, did not change significantly [19].

The commercially available AFib 2gether app was designed to support AFib patients who are not yet receiving OAC therapy in shared decision-making. The app was developed by cardiologists in collaboration with Pfizer. Patients answer questions via the app to determine their thromboembolism risk using the CHA_2_DS_2_-VASc score (congestive heart failure, hypertension, age ≥75 years [doubled], diabetes mellitus, prior stroke, or transient ischemic attack [doubled], vascular disease, age 65-74 years, sex category) and select questions to ask physicians. The information is transmitted to physicians. In addition, educational content is available. The protocol of a single-arm intervention study to assess usability and perceived usefulness has been published [23]. The usability categories of functionality and aesthetics were rated as 4.51 and 4.26 out of 5 by 37 patients and 4.19 and 4.04 out of 5 by 13 physicians, respectively, using the Mobile App Rating Scale [24]. Perceived usefulness was reported by patients with 40% to 62% agreement and by physicians with 59% to 82% agreement in three categories each. After the intervention, 12 out of 37 patients (32%) initiated OAC therapy. Patient involvement in the decision-making process was demonstrated in just under half (48%) of the 25 recorded consultations; additional face-to-face consultations were not conducted due to the COVID-19 pandemic. The study was sponsored by Pfizer.

### Smartphone Apps for Increasing Therapy Adherence

#### Platelet Inhibitors

Mobile4Meds is the protocol of an RCT comparing the ability of digital tools to increase adherence to antiplatelet therapy after acute coronary syndrome [25]. Prior to the RCT, the researchers assessed patients’ perceptions regarding text messaging and the use of two different apps: Medisafe and Mango Health, both of which are commercially available. Six focus groups were moderated by professionals and had a specific topic, such as the patient’s motivation to take their medication, presumed benefits and disadvantages of text messages and mobile apps, as well as the evaluation of the above-mentioned apps after testing each for a week [26]. Results showed that despite an average age of 66.9 years, all patients were regular smartphone users and described the use of mobile apps to enhance therapy adherence as useful. The reasons for nonadherence were forgetfulness and everyday distractions. Both text messaging and mobile apps were perceived to be able to address this issue effectively. Patients especially appreciated the interactive design of apps, the additional information about medication, the visualization of medication intake, and health parameters. One key finding was that most patients desired to share their results with their general practitioner, but data safety was a major issue. The project received funding from the National Institutes of Health (NIH), the University of California, and the US Department of Veterans Affairs.

A randomized feasibility study among 45 patients receiving dual antiplatelet therapy (DAPT) after percutaneous coronary intervention (PCI) over 3 months evaluated the impact of the mobile app MyIDEA (My Interventional Drug-Eluting Stent Educational App) on therapy adherence [27]. MyIDEA educates patients via short stories (ie, patient narratives). The authors discovered that patients show a high interest in smartphone-based support tools. However, they could not detect a significant difference in therapy adherence or anxiety levels between those who received traditional education materials and those who used the app. Limitations of the study were a small study population, a small local range, and low socioeconomic diversity.

The Me & My Heart app is not yet available in app stores, but the protocol of a prospective, randomized, multicenter, open-label interventional device study has been published regarding the app, which is CE (Conformité Européene) marked as a class I medical device [28]. It was developed by physicians and patients with sponsorship from AstraZeneca, the manufacturer of ticagrelor. The intended study compares two groups receiving DAPT with respect to medication adherence and lifestyle changes for 48 weeks after PCI. While both groups receive the same monthly evaluation questionnaires via the app, the intervention group also receives medication intake reminders, educational content, as well as motivational and supportive text messages. Therapy adherence is measured using either a self-developed questionnaire or a medication event monitoring system (MEMS) device. MEMS devices hold medication blister packs and register medication intake without displaying anything to the patient to minimize bias.

Wittig-Wells et al [29] described results from a prospective open-label trial evaluating the effects of a mobile app with reminders on adherence to antiplatelet therapy in patients after hip or knee alloplasty. There were 195 patients enrolled, and 122 completed the pill count at the end of the follow-up. No statistically significant differences were described between the two groups.

A prospective observational study conducted by Senoo et al [30] showed promising results in terms of adherence improvement in older adult patients with AFib. The Medisafe medication management app is a medication management platform, which includes the name of the medication and the dose. No detailed app description was provided. It has been underlined that mHealth technology, which emphasizes education, automatic reminders, and patient engagement, may be helpful. Additionally, reminders that do not require a doctor’s involvement are a significant tool, which could enhance everyday care.

#### Anticoagulation Therapy

The app AFib Connect was developed by physicians and designers to support self-care and therapy adherence in patients taking direct oral anticoagulants (DOACs) for thromboembolic prophylaxis in AFib. It provides verified information on AFib, treatment options, and stroke risk. The app offers tools to track episodes of AFib and to document possible triggers, it includes a heart rate monitor that uses the phone camera to enable self-documentation, and it sends reminders for appointments and medications. The app’s usability and usefulness were assessed by interviewing 12 patients with AFib [31]. The authors concluded with three key recommendations for successful app development: (1) understand your user group, (2) understand the users’ workflow, and (3) assess required changes over time. Daiichi Sankyo Inc, a pharmaceutical company that manufactures the DOAC edoxaban, sponsored the study.

Physicians from New York teamed up with software developers to report on the efficacy of AiCure, a commercially available app using artificial intelligence to monitor adherence to anticoagulant intake and to provide reminders and specific instructions [18]. A randomized study with 28 patients compared adherence to anticoagulants when using AiCure to adherence when no monitoring device was used. App users had higher adherence than nonusers based on plasma levels of anticoagulants (100% vs 50%) and pill count (97.2% vs 90.6%). The project was funded by the NIH and sponsored by AiCure, LLC.

### Smartphone Apps for Increasing Guideline Adherence and Decision Support

In a collaboration between Vanderbilt University and the American Society of Regional Anesthesia and Pain Medicine (ASRA), the ASRA Coags app was developed, which offers anesthesiologists decision support for the management of patients taking antithrombotic therapy and receiving regional anesthesia [32]. Users can choose from a broad variety of antithrombotic therapy, including antiplatelets and OACs; three different regional anesthesia procedures; and four different perioperative scenarios. Recommendations are based on the guidelines from the ASRA. An RCT with 259 anesthesiologists compared their performance with clinical scenarios related to ASRA guidelines, using either the ASRA Coags app; any other resource, such as the ASRA website; or no other resource. Participants using the ASRA Coags app gave significantly more correct answers than participants in the control group (mean 92.4%, SD 6.6% vs mean 68.0%, SD 15.8%; *P*<.001) [33]. ASRA Coags received grants from the Foundation for Anesthesia Education and Research for app development and from the Vanderbilt University Medical Center for the RCT [32,33]. After an update of the ASRA guidelines in April 2018, a study was conducted to evaluate how using the ASRA Coags app may facilitate guideline implementation. The authors stated that guideline alterations usually take about 17 years to be established in clinical use. Since 90% of all ASRA Coags users updated their apps within a month of the new guidelines being released, it has been postulated that mobile phone apps may be an effective tool to accelerate the implementation of new guidelines [34]. However, the authors had no information about whether the new information provided by the app was transferred to clinical use.

An unnamed app recommends OAC for patients with AFib based on CHA_2_DS_2_-VASc scores, according to US guidelines. Adherence to guidelines was compared among 10 cardiologists before and after the introduction of the app to 191 and 182 patients with newly diagnosed AFib, respectively. The use of OAC increased significantly from 37% to 51% (*P*=.01), and adherence to guidelines increased significantly from 46% to 66% (*P*<.001). The trial was funded by the authors’ institution [35].

The commercially available app Management of Anticoagulation in the Periprocedural Period (MAPPP) was developed by a multidisciplinary team and provides physicians with evidence-based guidance on perioperative management of OAC therapy. Recommendations are based on the patient’s medication and on the procedure’s bleeding and thromboembolic risk as assessed by the physician. Warfarin (41%) and rivaroxaban (24%) were the most frequently selected medications [36]. Almost half of the queries included high bleeding risk procedures (49%) or high thromboembolic risk procedures (45%). The combination of high bleeding and high thromboembolic risk accounted for 30% of all queries. Due to the study design, it is unknown whether or not the documented user episodes were used in patient care.

A subsequent study investigated the effect of the integration of the MAPPP app into the electronic health record of patients receiving OAC therapy. Two cohorts were defined by the physician’s decision to follow or decline the MAPPP app’s advice. The two groups were compared in a 30-day follow-up after admission to the emergency department. The study revealed that physicians are more likely to follow the guidelines provided by the app with younger patients with normal renal function and who are taking DOAC medication, whereas they tend to trust their clinical expertise and deviate from the guidelines with older patients with impaired renal function and who are receiving warfarin therapy. The study showed a significantly lower rate of admissions to the emergency department for the cohort in which health practitioners accepted the app’s advice than in the cohort where the advice was rejected (4.0% vs 8.3%; *P*=.02) [37].

The app PTT (Partial Thromboplastin Time) Advisor is a commercially available decision support tool for physicians with patients presenting with abnormal partial thromboplastin time and normal prothrombin time values. It recommends laboratory tests based on an algorithm created by the Centers for Disease Control and Prevention (CDC) [38]. The app Anticoagulation Manager succeeds PTT Advisor and provides multiple algorithms for patients with different conditions, such as venous thromboembolism or AFib. A user evaluation is planned. Anticoagulation Manager received funding from the CDC, the NIH, the Georgia Cancer Coalition, the Georgia Research Alliance, Hewlett-Packard Inc, and Microsoft Research [39].

A retrospective study on 274 patients with the indication for DOACs evaluated the support tool RecosDoc-MTeV, which was embedded in the hospital information system, alongside the Assistance Publique – Hôpitaux de Paris (AP-HP) clinical practice guidelines (CPGs) and a companion smartphone app, which was developed by the Parisian public hospitals [40]. Both clinical decision support systems analyzed the patient’s clinical information with closed questions or validated scores. The recommended treatment was compared with the one received during hospital admittance. Both tools were congruent with their recommendations in 96.7% of all cases, whereas the received treatments varied between 67.2% and 72.3% from the recommendations provided by RecosDoc-MTeV and the AP-HP CPGs, respectively. Whenever received treatments and clinical decision support systems were not aligned, both tools recommended the same treatment. One limitation of the study was that only the type of anticoagulant was analyzed, whereas neither dosage nor period of treatment were analyzed.

## Discussion

### Principal Findings

We identified four groups of apps within the 32 eligible publications returned by this scoping literature review: (1) apps for patients supporting self-management of VKAs, (2) apps for patients increasing therapy adherence, (3) educational apps for patients, and (4) apps for physicians in supporting guideline adherence. This illustrates that such apps offer broad possibilities. Overall, most apps address OAC therapy for the most common indication, AFib. It is to be emphasized that within industrialized countries, the most frequent medications for AFib are DOACs, a group of drugs with high economic impact [41]. Interestingly, few sources reported on app projects addressing patients receiving antiplatelet therapy, although this medication is taken regularly by one-third to half of the adult population over 65 years [42-45]. Also, studies reveal that managing DAPT is challenging for medical professionals [46].

When apps for patients are grouped by the type of antithrombotic therapy they focus on, it is noticeable that many of them focus on one particular drug (eg, warfarin) or drug subgroup (eg, DOACs), rather than offering a broad selection of different antithrombotic therapy drugs. On the one hand, this might be due to the popularity of warfarin, especially in lower-income countries [47]. On the other hand, many of these apps are sponsored by pharmaceutical companies distributing such drugs. Benefits to this narrow approach are that maintaining these apps may be more cost-effective and that their features are tailored to their users’ needs. The threat, however, is that medical apps are often known to be used as data collection mechanisms [48]. This is of high economic value to pharmaceutical companies. Data security is challenging for developers of medical apps [49]. The user may not be informed properly about the uses of collected data [50]. Also, sensitive personal and medical data are shared with third parties, potentially leading to serious damage to the user [50,51]. Antithrombotic therapy is a sensitive topic and, therefore, it is essential that medical staff and patients are provided with only the most up-to-date and precise information. Incorrect use of the medication might have life-threatening effects. There are also numerous apps for other indications (eg, type 1 diabetes, contraception, and more), but we have deliberately focused on antithrombotic therapy, as we are convinced that this topic, particularly in the perioperative setting, might bear a high risk of incorrect treatment decisions. In addition, most apps target diseases among younger patients, but this seems to overlook the fact that older adult patients are also increasingly using smartphones. Moreover, antithrombotic therapy management supported by computer-based technologies might increase the overall quality of treatment, translating into increased patient satisfaction [52].

User statistics from the MAPPP and the ASRA Coags apps show the need for high-quality and universal evaluation tools recommending periprocedural management of patients taking anticoagulants. They show that involvement of medical professionals as a quality predictor increases download numbers [53]. However, only 13% of medical apps targeting the general population have involved medical experts during development [54]. Yet, patients as medical laymen are hardly able to assess the quality of medical content. Publications on development and evaluation studies are one of the instruments potentially ensuring high-quality standards. This review revealed a relatively small number of scientific reports given the enormous number of apps in the app stores. Other instruments include compulsory or voluntary certifications that ensure quality standards for medical apps.

### Secondary Findings

Interestingly, from all 32 records, only one reported on apps that were both developed by European research groups and commercially available on the common app platforms, the Apple App Store and Google Play [40]. Currently, within the European Union, the registration of an app as “Medical Device Software” has become a financial and administrative obstacle [55]. This is critical for most nonprofit apps since most public funding provides no funds for legal certification after the research and development phase. This hurdle may also be partly responsible for the discrepancy between peer-reviewed apps developed by renowned institutions and their availability.

In this review, most commercially available apps with a scientific background were developed in countries with lower data security [56]. For example, the United States and China are leading the field of app research but only hold rank 21 and 69, respectively, in the National Cyber Security Index released by the Estonian government and funded by the European Union; it is of note that other institutions provide other rankings.

Another issue is the maintenance of a published app that will guarantee not only the latest medical information but that the diverse technical needs for different phones and software will be met. This issue has also been addressed by the developers of the Alfalfa app, who decided to use the WeChat platform to evade the above-mentioned technical maintenance issues and requirements by the Apple App Store and Google Play. However, WeChat has been widely criticized for their data protection policy and entwinement with the Chinese government [57,58].

To overcome these obstacles, app developers need to seek strong financial partners. We argue that this might lead to a potential disadvantage for users with rare diseases or less financially potent drugs. In addition, a medication- or indication-based approach neglects the fact that many patients have several conditions that may require different kinds of antithrombotic therapy. However, a one-size-fits-all solution requires continuous maintenance by specialists, which may be less attractive for commercial distributors.

### Strengths and Limitations

This scoping literature review provides extensive insight into the current state of scientific development and evaluation of medical apps that address antithrombotic therapy as their main topic. To our knowledge, this is the first review with this scope. We decided to focus on this medication because it also includes the obstacles of app development for older adults, is very frequently used, and offers a versatile spectrum of intended use for medical apps. Limitations of this study may be that the amount of information in the digital world is extensive, and a clear selection of data to enhance readability will always come at the cost of information loss. Taking into consideration the methodology, selection bias during full-text screening could occur since both inclusion and exclusion criteria leave some space for interpretation. On the other hand, this part was described in detail and, in case of any doubt, it was cleared by other authors. Moreover, it should be emphasized that a scoping review as such cannot show all the data, as this was not its objective. This review only targeted antithrombotic therapy apps with scientific evaluation, independently of the involvement of medical professionals or medical societies. Therefore, apps such as ManageAnticoag by the American College of Cardiology could not be included [59]. As already mentioned, AFib is the most common indication for anticoagulants, and there are several apps available for both patients and physicians concerning this indication. However, since the scope of this review was antithrombotic therapy and not AFib, publications focusing on AFib were not included, but they were reviewed elsewhere recently [60,61]. Therefore, papers that seemed relevant to the field at first sight were not included. Additionally, we only focused on apps that could be used by people where additional devices were not needed, which was an exclusion criterion but significantly limited the number of apps described.

### Future Directions

Although the app market is constantly growing, only few solutions concerning antithrombotic therapy have been developed and evaluated following a scientific approach. In addition, the current legal situation might not facilitate independent, patient-orientated, and thoroughly validated apps. Despite diet management being a crucial aspect for patients receiving VKA therapy, no scientific records regarding such apps were retrieved by our search. The focus of most of the existing mobile phone apps is rather narrow, creating a need for many different apps. The desire for a certified and comprehensive solution seems unaddressed. Therefore, we propose the development of an app that has a wide target group, is based on scientific guidelines, and has supporting medical evidence and adequate data security. It should provide users with validated educational content, which will be constantly updated. Ideally, this app should address patients and doctors alike to create a common base for shared decision-making. Nonetheless, these solutions require further evaluation studies, which should be carried out prior to the commercial distribution of the app.

The approach to use apps to ameliorate clinical management and adherence to treatment is relatively new. Nevertheless, we see a great need for such instruments for both patients and physicians. Therefore, we advocate for more large prospective studies since we noticed many trial protocols but very few implementations. Positive results from studies could lead to broader inclusion in international guidelines. Our paper should be also regarded as a call for balance between studies and market access.

## Appendix

Multimedia Appendix 1 Detailed search strategy.

Multimedia Appendix 2 Synthesis of results.

## Abbreviations

AFib: atrial fibrillation
AP-HP: Assistance Publique – Hôpitaux de Paris
ASRA: American Society of Regional Anesthesia and Pain Medicine
CDC: Centers for Disease Control and Prevention
CE: Conformité Européene
CHA_2_DS_2_-VASc: congestive heart failure, hypertension, age ≥75 years (doubled), diabetes mellitus, prior stroke, or transient ischemic attack (doubled), vascular disease, age 65-74 years, sex category
CPG: clinical practice guideline
DAPT: dual antiplatelet therapy
DOAC: direct oral anticoagulant
INR: international normalized ratio
MAPPP: Management of Anticoagulation in the Periprocedural Period
MASS: Mobile Applications for Seniors to Enhance Safe Anticoagulation Therapy
MEMS: medication event monitoring system
mHealth: mobile health
MyIDEA: My Interventional Drug-Eluting Stent Educational App
NIH: National Institutes of Health
OAC: oral anticoagulation
PCI: percutaneous coronary intervention
PDA: patient decision aid
PTT: Partial Thromboplastin Time
RCT: randomized controlled trial
VKA: vitamin K antagonist
