# Supplementary material for: Smartphone Apps for Managing Antithrombotic Therapy: Scoping Literature Review
Source: JMIR Cardio. 2022 Jun 21;6(1):e29481. doi: 10.2196/29481 (PMC9257616; doi:10.2196/29481)
Supplement: Multimedia Appendix 1 [file cardio_v6i1e29481_app1.docx]

**Multimedia Appendix 1: Detailed search strategy**

**MEDLINE via PubMed (Last Searched 25/01/2022)**

**Textwords:**

| Search | Search terms |
| --- | --- |
| 1 | anticoagulant* [tiab] |
| 2 | antiplatelet [tiab] |
| 3 | anti platelet [tiab] |
| 4 | antithrombotic [tiab] |
| 5 | anti thrombotic [tiab] |
| 6 | smartphone [tiab] |
| 7 | smartphone app* [tiab] |
| 8 | mobile app* [tiab] |
| 9 | ((((#1) OR (#2)) OR (#3)) OR (#4)) OR (#5) |
| 10 | ((#6) OR (#7)) OR (#8) |
| 11 | (#9 AND #10) |

**MESH-Terms:**

| Search | Search terms |
| --- | --- |
| 12 | "Mobile Applications"[Mesh] |
| 13 | "Smartphone"[Mesh] |
| 14 | "Anticoagulants"[Mesh] |
| 15 | “Platelet Aggregation Inhibitors"[Mesh] |
| 16 | (#12) OR (#13) |
| 17 | (#14) OR (#15) |
| 18 | (#16) AND (#17) |

**MEDLINE via Ovid (Last Searched 25/01/2022)**

**MeSH-Terms:**

| Search | Search term |
| --- | --- |
| 1 | Mobile Applications/ |
| 2 | smartphone/ |
| 3 | anticoagulants/ |
| 4 | platelet aggregation inhibitors/ |
| 5 | 1 or 2 |
| 6 | 3 or 4 |
| 7 | 5 and 6 |

**Textwords:**

| Search | Search term |
| --- | --- |
| 8 | anticoagulant*.ti,ab. |
| 9 | antiplatelet.ti,ab. |
| 10 | anti platelet.ti,ab. |
| 11 | antithrombotic.ti,ab. |
| 12 | anti thrombotic.ti,ab. |
| 13 | smartphone.ti,ab. |
| 14 | smartphone app*.ti,ab. |
| 15 | mobile app*.ti,ab. |
| 16 | 8 or 9 or 10 or 11 or 12 |
| 17 | 13 or 14 or 15 |
| 18 | 16 and 17 |
