# Supplementary material for: Smartphone Apps for Managing Antithrombotic Therapy: Scoping Literature Review
Source: JMIR Cardio. 2022 Jun 21;6(1):e29481. doi: 10.2196/29481 (PMC9257616; doi:10.2196/29481)
Supplement: Multimedia Appendix 2 [file cardio_v6i1e29481_app2.docx]

Multimedia Appendix 2: result synthesis

Overview of the 32 included records stating the type of record, name of the reported app, commercial availability of the reported app (based on a self-conducted search in the Android Play Store and Apple App Store; apps marked with * are commercially available on other platforms), the target group of the app, the aim of the app and the main results reported, funding and country of origin of the app as reported in the records.

| **Record** | **Type** | **App** | **Commercially available?** | **Target group of app** | **Aim of app** | **Main result** | **Funding** | **Country** |
| --- | --- | --- | --- | --- | --- | --- | --- | --- |
| **Group 1: Smartphone apps for patients managing VKA therapy** | | | | | | | |  |
| [8] | development of app | Warfarin Guide | yes | Patients on Warfarin | management of Warfarin therapy / dosage recommendation according to INR | co-design with developers and patients enables user friendliness and addresses user needs | n.a. | Norway |
| [10] | evaluation | Warfarin Guide | yes | Patients on Warfarin | management of Warfarin therapy / dosage recommendation according to INR | successful development via user-centered design approach; providing flexibility and decision support; further validation planned (RCT) | n.a. | Norway |
| [11] | development of app; evaluation | Not named | unknown | Patients on VKA; physicians | tracking INR measurements and recommending dosages; communication with physicians | technical implementation was successful; dosage recommendations could be validated in a mini-trial | n.a. | Germany |
| [12] | efficacy testing | Anticlot Assistant | no | patients on warfarin; physicians | decision support function based on target and current INR | Compliance was generally low; TTR was significantly better in patients with high compliance compared to low compliance | China National Natural Science Foundation | China |
| [13] | study protocol | XY app | no | Patients on warfarin; health care professionals | home management of Warfarin therapy with dosage recommendation according to INR | TTR and quality of life will be compared to a control group in an RCT | China Medical Board | China |
| [14] | development of app; evaluation | Alfalfa | no* | Patients on warfarin; health care professionals | home management of Warfarin therapy with dosage recommendation according to INR, patient education | usability has higher requirements for patients and learnability was generally low. | n.a. | China |
| [15] | efficacy testing | Alfalfa | no* | Patients on warfarin; health care professionals | home management of Warfarin therapy with dosage recommendation according to INR, patient education | the app group was superior to a control group concerning TTR, major bleeding events, warfarin-related emergency department visits and hospital admissions in a retrospective study | Natural Science Foundation of Fujian Province of China | China |
| [16] | efficacy testing | YiXing | no | Patients on warfarin; clinical pharmacists | home management of Warfarin therapy with dosage recommendation by clinical pharmacists according to INR, patient education | patient’s awareness and days within TTR were increased in app users but no influence on correct-warfarin-taken-days or the incidence of anticoagulation related complications was detected | Guangdong Basic and  Applied Basic Research Foundation, Young Teacher Foundation of Sun Yat-Sen University, National Key R&D Program of China, Special fund for clinical research of  Wu Jieping medical foundation | China |
| **Group 2: Smartphone apps for educating patients** | | | | | | | |  |
| [17] | review | various | unknow | patients on VKA therapy | provide education and improve adherence | positive impact on patient’s knowledge, further studies required | National Research Foundation of Korea | Korea |
| [20] | development of app; efficacy testing | Not named | unknown | AFib patients on OAC | facilitate the shared decision making on thromboembolic prophylaxis in AFib; to be used during medical visits | knowledge was significantly increased; self perception of risks did not significantly change | internal | Brazil |
| [22] | development of app | MASS | no | elderly patients on Warfarin | provide education and management modules | elderly patients claim for mHealth solutions for the management of warfarin medication; user needs can be assessed by semi-structured interviews | university grants | USA |
| [19] | efficacy testing | MASS | no | elderly patients on Warfarin | provide education and management modules | OAC knowledge significantly improved after 3 months of usage | university grants | USA |
| [21] | development of app; evaluation; efficacy testing | not named | unknown | AFib patients | facilitate shared decision making on thromboembolic prophylaxis in AFib; to be used during medical visits | knowledge was significantly increased; decisional conflict was significantly decreased; acceptability was good | Industrial (Pfizer) | Philippines |
| [23] | study protocol | AFib 2gether | yes | AFib patients without OAC | educate patients to facilitate shared decision making on using OAC; to be used prior or during medical visits | usability, perceived usefulness, impact on shared decision making and start of OAC will be evaluated in a single arm study | Industrial (Pfizer) | USA |
| [24] | evaluation | AFib 2gether | yes | AFib patients without OAC | educate patients to facilitate shared decision making on using OAC; to be used prior or during medical visits | usability and usefulness was rated high; involvement of patients in decision making was proven; intervention led to start of OAC in one third of cases | Industrial (Pfizer) | USA |
| **Group 3: Smartphone apps for increasing therapy adherence** | | | | | | | |  |
| [25] | study protocol | various | yes | ACS/PCI patients on antiplatelets | improve adherence to antiplatelet medications after ACS or PCI | Adherence to medication in app users will be compared to text messaging and a control group in an RCT | San Francisco Veterans Affairs Medical Center | USA |
| [26] | evaluation | various | yes | ACS/PCI patients on antiplatelets | improve adherence to antiplatelet medications after ACS or PCI | Apps were perceived as convenient and helpful especially features like interactivity, individualized health monitoring, and personalized information | San Francisco Veterans Affairs Medical Center | USA |
| [27] | evaluation; efficacy testing | myIDEA | no | patients on DAPT | Increase adherence to DAPT treatment through patient education | MyIDEA was used in hospital and outpatient setting; no significant difference in adherence between intervention and control | university grants | USA |
| [28] | Study protocol | Me & My Heart | no | patients on DAPT with ticagrelor | Increase adherence to medication | Patients were randomized to intervention or control group | Industrial (AstraZeneca) | Germany |
| [29] | efficacy testing | The Medisafe Medication Management | yes | patients after hip or knee replacement with prescribed aspirin | Increase adherence to medication | No differences in adherence between standard care group and app augmented group | Multiyear research grant from the National Association of Orthopaedic Nurses (NAON) | USA |
| [30] | efficacy testing | Smart AF | no | elderly patients with AFib on anticoagulation | improve medication adherence in elderly patients with AFib | improved medication adherence among elderly patients with AFib | industrial (Bristol Myers Squibb/ Pfizer Japan Thrombosis Investigator Initiated Research Program) | Japan |
| [31] | evaluation | AFib Connect | no | AFib patients on DOACs | supporting long-term patient self-care and adherence to anticoagulant therapy | app usability and usefulness were rated high in this pilot study | industrial (Daiichi Sankyo) | USA |
| [18] | efficacy testing | AiCure | yes | patients on OAC | increase adherence to OAC treatment | AI platform patients had higher adherence by visual and laboratory testing | National Center for Advancing Translational Sciences; industrial (AiCure) | USA |
| **Group 4: Smartphone apps for increasing guideline adherence and decision support** | | | | | | | |  |
| [32] | download / user report | ASRA Coags (Regional) | yes | anaesthesiologists | provide recommendations on managment of antithrombotic therapy in patients requiring regional anaesthesia | rivaroxaban, clopidogrel and prophylactic enoxaparin were top three medication; 78% were neuraxial blocks | Foundation for Anesthesia Education and Research | USA |
| [33] | efficacy testing | ASRA Coags | yes | anaesthesiologists | increase application of ASRA guidelines | in this RCT users of ASRA Coags performed significantly better in a test of knowledge of the ASRA guidelines | grant from Vanderbilt Institute for Clinical and Translational Research | USA |
| [34] | efficacy testing | ASRA Coags | yes | anaesthesiologists | evaluate time until users  update their phones after a new release | after 7 and 30  days 80%  and 90% of the users , respectivly, updated their ASRA Coags app with the new guidelines. | No funding declared | USA |
| [35] | efficacy testing | Not named | unknown | cardiologists | improve the adherence to AHA/ACC 2014 guidelines for the management of patients with AF by proposing recommendation based on CHA2DS2-VASc and HAS-BLED | adherence to anticoagulation guideline for the treatment of AFib was significantly improved after the intervention | Mashhad University of Medical Sciences | Iran |
| [36] | development of app; download / user report | MAPPP | yes | physicians | increase access to current expert guidance on periprocedural anticoagulation management | successful dissemination; no provider and patient specific data | Centers for Medicare & Medicaid Services (CMS)  US Department of Health and Human Services | USA |
| [37] | efficacy testing | MAPPP | yes | physicians | increase access to current expert guidance on periprocedural anticoagulation management and improve patient safety | Acceptance of the app‘s recommendation was significantly associated with fewer ED visits within 30 days postprocedure | Industrial (Janssen, Boehringer Ingelheim);  Centers for Medicare & Medicaid Services (CMS)  US Department of Health and Human Services; Broxmeyer Fellowship in Clinical Thrombosis | USA |
| [38] | development of app | PTT Advisor | yes | physicians | providing an electronic, interactive decision support tool to select appropriate tests for patients with prolonged PTT and normal PT/INR | UI and UX are critical aspects during app development and should be addressed by experts | n.a. | USA |
| [39] | development of app | Anticoagulation Manager (further development of PTT Advisor) | yes | physicians | provide guidance to prescribe the most appropriate anticoagulant drug and dosage based on laboratory tests | Multiple scenarios and indications are included in the algorithm | US Department of Health and Human Services | USA |
| [40] | Development of app; efficacy testing | RecosDoc-MTeV  AP-HP app | yes | physicians | Support guideline-based decision making for anticoagulation therapy or prevention in venous thrombembolism | Both apps showed highly consistent recommendations; good compliance to recommendations was shown in a retrospective evaluation | n.a. | France |
| Abbreviation: ACS (acute coronary syndrome), ACC (American College of Cardiology), AFib (atrial fibrillation), AHA (American Heart Association), AI (artificial intelligence), ASRA (American Society of Regional Anesthesia and Pain Medicine), DAPT (dual antiplatelet therapy), DOAC (direct oral anticoagulants), ED (emergency department), INR (international normalized ratio), MAPPP (Management of Anticoagulation in the Peri-​Procedural Period app), MASS (Mobile Applications for Seniors to enhance Safe anticoagulation therapy), OAC (oral anticoagulants), PCI (percutaneous coronary intervention), PT (prothrombin time), PTT (partial thromboplastin time), RCT (randomized controlled trial), TTR (time in therapeutic range), UI (user interface), UX (user experience), VKA (vitamin K antagonist) | | | | | | | | |
